# Food-Borne Nanocarriers for Calcium Delivery: A New Choice for Nutrient Supplements

**DOI:** 10.3390/foods11030308

**Published:** 2022-01-24

**Authors:** Nanying Wang, Yannan Chen, Yukun Song, Deyang Yu, Mingqian Tan

**Affiliations:** 1 Academy of Food Interdisciplinary Science, School of Food Science and Technology, Dalian Polytechnic University, Qinggongyuan1, Gangjingzi District, Dalian 116034, China; louis493942754@gmail.com (N.W.); 18954530681@163.com (Y.C.); songyukun@126.com (Y.S.); yudy0411@163.com (D.Y.); 2 National Engineering Research Center of Seafood, Dalian Polytechnic University, Dalian 116034, China; 3 Collaborative Innovation Center of Seafood Deep Processing, Dalian Polytechnic University, Dalian 116034, China

**Keywords:** beef, one-step hydrothermal, food-borne nanoparticles, calcium binding, nutrient supplements

## Abstract

Calcium is considered as an important nutrient element for the maintenance of human health, and food-borne nanoparticles (FNs) produced during food processing may have potential as nanocarriers for calcium ion delivery. Beef is an important source of animal protein that has high protein and low fat content and is rich in a variety of amino acids; thus, beef may be a suitable material for the development of calcium nanocarriers. In this paper, FNs were synthesized from beef by one-step hydrothermal synthesis. The FNs had a spherical shape with a size of about 3.0 nm and emitted a bright blue fluorescence under 365 nm ultraviolet irradiation. The amino nitrogen atom and carboxyl oxygen atom of the functional groups on the surface of the FNs were the main binding sites for the chelation of Ca(II). The size of the FNs-Ca(II) complex was about 4.75 nm, and the specific signal peak of calcium at 3.7 keV was observed in its energy dispersive X-ray spectroscopy spectrum. The viability of cells treated with FNs-Ca(II) was more than 65%, while viability was only 60% after treatment with CaCl_2_. The results showed that the FNs from beef have great potential in calcium delivery for the development of a calcium supplement.

## 1. Introduction

Calcium has been regarded as an essential nutrient element in life [1]; it has many roles in metabolism and life maintenance and is closely related to several functions, such as immunity, nerve transmission, digestion, and circulation [2]. Many physiological diseases, such as bone and tooth dysplasia in children [3], cramps and pregnancy hypertension syndrome in women [4,5], and osteoporosis and fracture in the elderly, are caused by a lack of calcium [6,7]. Therefore, the health problems caused by calcium deficiency should arouse enough attention to promote the essential development of innovative calcium supplements to overcome calcium deficiency diseases.

Inorganic calcium salts, such as calcium carbonate, are the most common calcium supplement because of their high calcium content [8]. However, in the absorption process, a large amount of gastric acid will be consumed [9], resulting in an increased risk of kidney stones [10]. Fructooligosaccharides can provide calcium with better water solubility, less intestinal stimulation, and higher bioavailability; however, the calcium content is significantly lower than that in inorganic calcium salt [11]. In recent years, peptides from food-grade protein that can chelate calcium ions have been developed as new calcium supplements. For instance, Mellander et al. hydrolysed milk with trypsin and isolated casein phosphopeptides (CPPS) with calcium-chelating ability [12]. Zhao et al. reported on a peptide in the hydrolysate of Pacific cod skins with a calcium-binding ability [13]. Sun et al. described a heptapeptide from the ovum of sea cucumber as a chelating agent for enhanced calcium delivery [14]. Although these peptides have excellent calcium chelating abilities, the process of preparing the peptides is tedious and costly, which makes it difficult to be widely used [15]. Therefore, the development of an innovative calcium carrier with a simple production process, higher stability, and better biocompatibility is highly desirable. In recent years, food-borne nanoparticles (FNs) from our daily foods have attracted wide attention due to their advantages, including their low cost, easy attainability, good water solubility, excellent optical performance, and good biocompatibility [16,17,18,19]. Zhou et al. found monodisperse high fluorescence FNs with a size of about 3 nm by the hydrothermal treatment of milk, which is a product consumed in daily life [20]. Song et al. reported nanoparticles extracted from roast sturgeon that could be used as nanocarriers for Fe(II) delivery [21]. Geng et al. reported that water-soluble FNs derived from beef broth treated by a pressure-cooker for 70 min have a potential application as nanocarriers for zinc [22]. It can be seen that FNs are ubiquitous in daily foods, and they have abundant functional groups on their surface, high water solubility, and an ultra-small size. Therefore, it is reasonable to assume that it may be an innovative idea to use FNs as a calcium carrier.

Herein, the FNs from beef were prepared by the hydrothermal treatment of beef, and the formation of FNs-Ca(II) complexes were fully characterized. Beef was selected as the raw material for the preparation of calcium nanocarriers because of its high protein content, low fat level, and rich variety of amino acids that are easily available in animal protein. The binding sites were analysed using spectrographic techniques. Finally, the cytotoxicity and biodistribution of FNs-Ca(II) was examined in Caco-2 cells using FNs as a control. Our data indicate that FNs with multifunctional groups have the potential to be used as carriers for calcium delivery.

## 2. Materials and Methods

### 2.1. Materials

Beef (short plate) was procured from the Qianhe market of agricultural products in Dalian, China. Anhydrous calcium chloride, potassium bromide, and other chemicals in this study were supplied by local reagent vendors. The Annexin V FITC/PI staining apoptosis kit was bought from Nanjing Jiancheng Bioengineering Institute.

### 2.2. Synthesis and Purification of FNs

At first, the beef (5 g) was crushed into paste by a meat grinder. The paste and deionized water (15 mL) were added to a 20 mL Teflon-lined autoclave which was then treated in an oven at 180 °C for 4 h. The suspension was poured out, centrifuged at 8000 rpm for 10 min, and the yellow filtrate was collected. Subsequently, the same volume of ethyl acetate was added to extract five times to remove the hydrophobic constituents. The fluorescent fractions were then collected by a Sephadex G-25 column with deionized water as an eluent. After they were concentrated and lyophilized, the FNs were finally obtained and stored at −80 °C for later use.

### 2.3. Preparation of the FNs-Ca(II) Complex

A total of 0.5 g of FNs were dispersed in 10 mL of sodium phosphate buffer (20 mM) at pH 7.8, heated in a water bath at 50 °C for 5 min, and then 1.5 g of CaCl_2_ were added. The FNs-Ca(II) complex was formed at 50 °C for 30 min under magnetic stirring. Thereafter, ultrafiltration (3000 Da) was used to centrifuge the FNs-Ca(II) complex at 4 °C, with a speed of 8000 rpm for 10 min. The supernatant was collected using a molecular weight cut-off of 3000 Da to remove the unbound calcium ions. After lyophilization, the FNs-Ca(II) complex was collected as a white powder.

### 2.4. FN and FNs-Ca(II) Complex Analysis

The shape and size of the prepared FNs and FNs-Ca(II) were characterized by transmission electron microscopy (TEM) using a JEM-2100 from JEOL Ltd. (Tokyo, Japan). A Lambda 35 spectrophotometer (PerkinElmer, Waltham, MA, USA) was used for the analysis of the ultraviolet-visible (UV-Vis) spectra of FNs and FNs-Ca(II). Fluorescence spectra were analysed by a F-2700 fluorescence spectrometer (Hitachi, Tokyo, Japan). An FLS 980-spectrofluorometer from Edinburgh Instruments (Edinburgh, UK) was used to measure lifetime with a 320 nm laser as the excitation source. X-ray diffraction (XRD), X-ray photoelectron spectroscopy (XPS), Fourier transform infrared spectroscopy (FT-IR), and zeta potential analysis were conducted using previously reported methods [15]. The microstructure and local element composition of the FNs and FNs-Ca(II) were characterized by a JSM-7800F scanning electron microscope from JEOL Ltd. (Tokyo, Japan). The ^1^H NMR spectra were obtained using a Bruker AV-400 analyser (Daltonics, Germany) with dimethyl-d_6_ sulfoxide as the solvent.

### 2.5. Calcium-Binding Capacity Assay

FNs were dispersed in deionized water at a concentration of 3 mg mL^−1^ and different masses of CaCl_2_ were added to the solution to achieve the mass ratios of CaCl_2_ to FNs of 1:3, 1:2, 1:1, 2:1 and 3:1. All testing samples were continuously stirred at 50 °C for 30 min, and then transferred to a dialysis bag (500 Da) to remove free calcium for 48 h. Finally, the volume of all samples was adjusted to 3 mL and the binding capacity of the FNs for calcium at different mass ratios was measured by flame atomic absorption spectrometry (Hitachi, Kyoto, Japan). 

### 2.6. Cytotoxicity and Biodistribution Analysis of FNs-Ca(II) in Caco-2 Cells

The Caco-2 cells bought from the Shanghai Institute of Biological Sciences, Chinese Academy of Sciences (Shanghai, China) were cultured in an MEM medium containing 1% penicillin-streptomycin and 20% fetal bovine serum and incubated at 37 °C in a 5% CO_2_ humidified atmosphere. The potential toxicity of the FNs, FNs-Ca(II), and CaCl_2_ to Caco-2 cells was detected using an MTT assay [16]. First, the Caco-2 cells were added into 96-well plates at a density of 1 × 10^5^ cells per well and incubated for 24 h. After removal of the medium, 100 μL of the samples with different concentrations (0.5, 1.0, 1.5, 2.0, 2.5, and 3.0 mg mL^−1^) were added and cultured for another 24 h. Then, 20 μL of MTT at a final concentration of 2.5 mg mL^−1^ was added into each well for 4 h at 37 °C. Later, the supernatant was carefully removed and 100 μL of dimethylsulfoxide solution was added. The cell viability was evaluated by measuring the absorbance at 570 nm. Annexin V-FITC/PI staining in a flow cytometer from BD (Franklin Lakes, NJ, USA) was used to measure the apoptosis and necrosis of Caco-2 cells. The distribution of FNs-Ca(II) (1 mg mL^−1^) was determined by a confocal laser fluorescence microscope (SP8 model, Leica, Wetzlar, Germany).

## 3. Results and Discussion

### 3.1. TEM Analysis of FNs-Ca(II)

**Figure 1:**
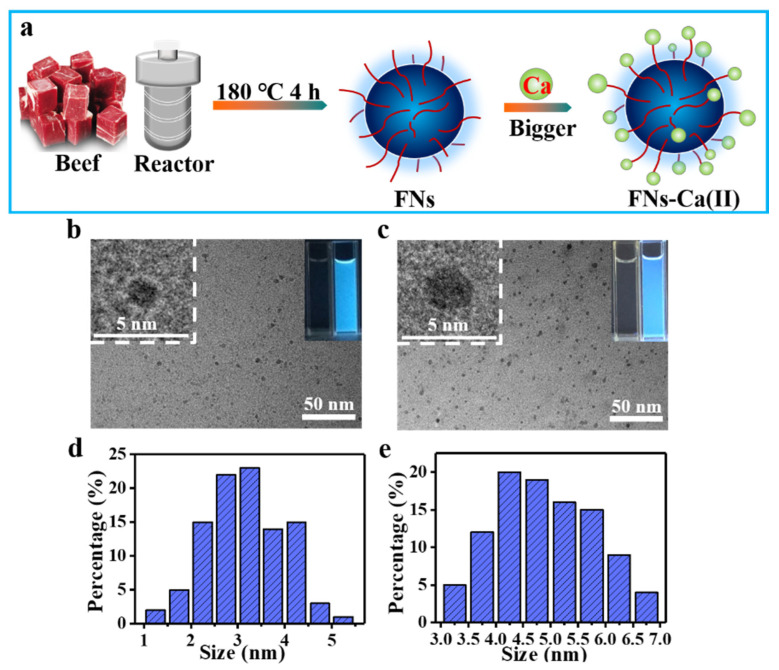
(**a**) Schematic illustration showing the beef FNs binding with calcium ions (FNs-Ca(II)). TEM images of FNs (**b**) and the FNs-Ca(II) complexes (**c**); insets represent high-resolution TEM images and the fluorescent photographs of water (left) and FNs/FNs-Ca(II) (right) under a UV lamp. Size distribution histogram for FNs (**d**) and FNs-Ca(II) (**e**).

The FNs synthesized from beef using a one-step hydrothermal synthesis method at 180 °C for 4 h were used as nanocarriers for Ca(II) (Figure 1a). As shown in Figure 1, the TEM images indicated that the FNs and FNs-Ca(II) were nearly spherical and uniformly dispersed (Figure 1b,c). The size distribution range of the beef FNs was about 3.0 nm, based on the calculations of a random selection of 100 particles (Figure 1d). Under high temperature and pressure, the proteins, lipids, and other substances in beef were broken down and condensed into small size FNs [23]. On the other hand, the size distribution range of FNs-Ca(II) was 3.0–7.0 nm, with an average size of 4.75 nm (Figure 1e). The size of FNs-Ca(II) was significantly larger than that of the FNs. The reason was possibly due to the interaction between the FNs and calcium after the FNs had chelated Ca(II). Similar results were also found in our previous studies [15]. In addition, the aqueous solutions of the FNs and FNs-Ca(II) were colourless and transparent; however, they exhibited blue fluorescence (Figure 1b,c, inset) under the illumination of a 365 nm ultraviolet light, which was consistent with the FNs from other foods [18]. The calcium-binding capacity of the FNs measured by flame atomic absorption spectrometry (Figure S1a) showed that with the increase in the proportion of calcium ions, the calcium-binding capacity also increased. When the mass ratio of calcium to FNs was 3:1, the binding ability was 3.5 times than that of the 1:3 ratio. This indicated that there were multiple binding sites on the surface of the FNs for calcium ions. Therefore, the mass ratio of 3:1 for calcium to FNs was selected and tested in the following experiments.

### 3.2. Optical Property Analysis of FNs and FNs-Ca(II)

**Figure 2:**
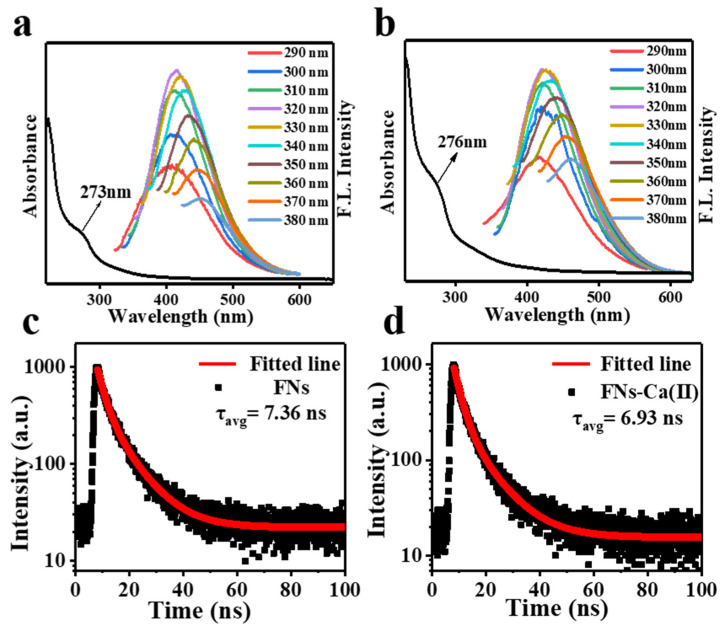
Fluorescence emission spectra and UV-Vis absorption spectra of FNs (**a**) and FNs-Ca(II) (**b**). Time-resolved fluorescence decay curve of FNs (**c**) and FNs-Ca(II) (**d**).

The optical property analysis showed that the FNs and FNs-Ca(II) complexes exhibited excitation-dependent fluorescence emissions, which were located at 415 nm and 421 nm, respectively (Figure 2a,b). The emission peaks of the FNs and FNs-Ca(II) complexes had a redshift. This was probably due to the different surface emission trap induced by the various functional groups present on the FNs [24]. In addition, the UV-Vis spectra showed a shoulder absorption peak in the region of 270–300 nm for both FNs and FNs-Ca(II), which was caused by the π→π* electron transition [25]. The absorption peak of FNs-Ca(II) increased from 273 to 276 nm due to the electron transfer transition from chelated FNs to calcium ions in the ultraviolet region [26]. Moreover, the fluorescence lifetimes (τ) of the FNs and FNs-Ca(II) were 7.36 and 6.93 ns (Figure 2c,d), respectively. This result was similar to that reported in the study of zinc nanocarriers from beef broth [23]. All the results indicated that the nanocarriers from beef FNs had unique fluorescence, which could be used in trace analysis in organisms.

### 3.3. XRD and FT-IR Analysis

**Figure 3:**
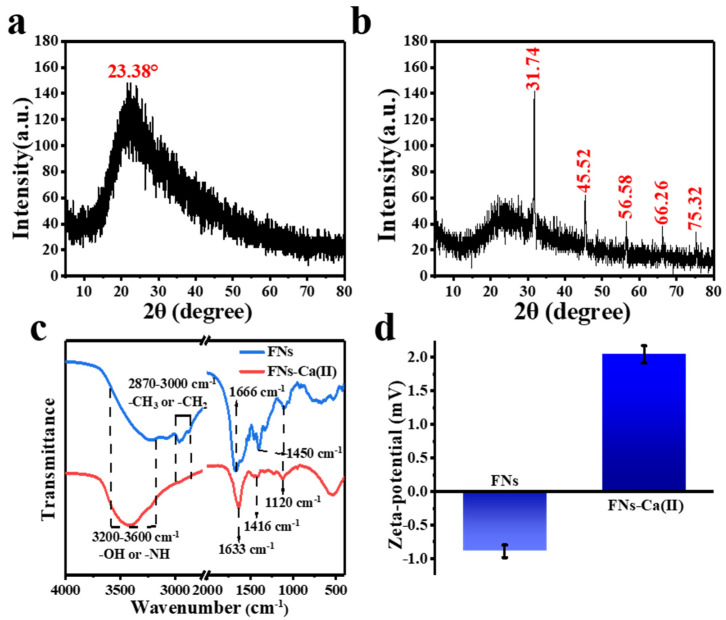
XRD pattern of (**a**) FNs and (**b**) FNs-Ca(II). (**c**) FT-IR spectra. (**d**) The zeta potential of FNs and FNs-Ca(II).

The ability and optical properties of FNs to interact with calcium ions may be due to the role of the surface functional groups of FNs. The XRD analysis of FNs (Figure 3a) showed that there was a wide diffraction peak at 2θ = 23.38°, which indicated the amorphous carbon property of the FNs. Our previous work about FNs obtained from roast pork had the same results [27]. Nevertheless, the XRD analysis of FNs-Ca(II) (Figure 3b) exhibites a strong peak at 2θ = 31.74° and some sharp peaks at 2θ = 45.53°, 56.58°, 66.26° and 75.32°. Compared with that of FNs, the peaks of FNs-Ca(II) were narrow and sharper, suggesting a crystallinity nature of the FNs-Ca(II) complex. The XRD results evidently suggested that calcium ions were chelated on the FN ligands because of the presence of sharp peaks originating from the complex of FNs and calcium [28]. The FNs’ FT-IR spectra and that of FNs-Ca(II) (Figure 3c) showed a clear shift of the FT-IR peaks for FNs-Ca(II), indicating the binding sites of the FNs and calcium ions. The absorption peak of both the FNs and FNs-Ca(II) at 3600–3200 cm^−1^ was ascribed to -OH and -NH stretching vibrations. With the coordination of calcium ions, the absorption band at 3228 cm^−1^ in the FNs spectrum changed to 3424 cm^−1^ in the FNs-Ca(II) spectrum, which indicated that an -NH group (hydrogen bond) had participated in the bonding and formed a Ca-N bond [29]. With the coordination of calcium, the vibration peak of the carboxyl group of FNs changed from 1666 cm^−1^ to 1633 cm^−1^, which suggested that the carboxyl group (COO-) was involved in the chelation with calcium. Besides, the vibration bands at 1044 cm^−1^ shifted to 1016 cm^−1^, suggesting that the calcium ions were bound to the carboxyl groups in FNs as well. Therefore, it was hypothesized that the binding sites of calcium and FNs were mainly located in the amino and carboxyl groups, which was consistent with other metal-binding FNs [15,22]. Moreover, the zeta potential can reflect the nanoparticle alteration, and the binding process may result in the change of zeta potential values. As shown in Figure 3d, the average potential of the FNs was −0.89 mV. After chelation with Ca(II) the electronegative value increased to 2.04 mV. The charge on the surface of the FNs decreased significantly, indicating that the FNs and calcium ions formed a new FNs-Ca(II) complex.

### 3.4. XPS Analysis of FNs and FNs-Ca(II) 

**Figure 4:**
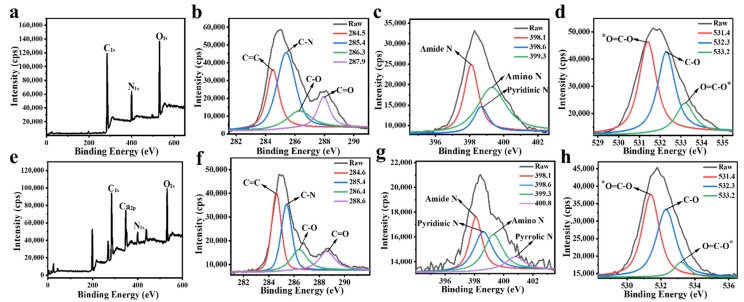
XPS survey of FNs (**a**) and FNs-Ca(II) (**e**). High-resolution XPS spectra of C_1s_ (**b**), N_1s_ (**c**), and O_1s_ (**d**) for FNs, and high-resolution XPS spectra of C_1s_ (**f**), N_1s_ (**g**), and O_1s_ (**h**) for FNs-Ca(II).

The XPS analysis was performed to further investigate the structure information and surface elemental composition of the FNs and FNs-Ca(II). Signal peaks of C_1s_ (285.8 eV), N_1s_ (399.6 eV), and O_1s_ (529.8 eV) for FNs were observed (Figure 4a), and the FNs were composed of carbon (66.38%), nitrogen (12.90%), and oxygen (20.72%). These elements were produced from the lipids, proteins, and carbohydrates in raw beef [16]. More specifically, the peaks at 284.5, 285.4, 286.3, and 287.9 eV, displayed in Figure 4b, confirmed the existence of C=C, C–N, C–O, and C=O chemical bonds, respectively. Figure 4c shows the high-resolution peaks of amide-N at 398.1 eV, pyridinic-N at 398.6 eV, and amino-N at 399.3 eV in the N_1s_ spectrum. The O_1s_ XPS spectra of FNs (Figure 4d) was decomposed into characteristic peaks at 531.4, 532.3, and 533.2 eV, demonstrating the existence of *O=C–O, C–O, and O=C–O* bonds, respectively [30]. Notably, the XPS results were in accordance with the FT-IR results. It was confirmed that the FNs had hydroxyl, amino, and carboxyl groups on their surface. Based on this, the FNs were a good choice for calcium ion coordination. In addition, the XPS spectrum of FNs-Ca(II) (Figure 4e) showed the specific signal peak of Ca(II) with a binding energy of 347 eV (Ca_2p_). The elemental ratios of C, N, O, and Ca in FNs-Ca(II) were 64.75%, 7.37%, 18.73%, and 9.14%, respectively. It was noteworthy that from the high-resolution N_1s_ spectrum of FNs-Ca(II), a new peak representative of Ca-N at 400.8 eV was observed (Figure 4g), indicating that calcium was successfully bonded with the beef FNs [31].

### 3.5. EDS Analysis of FNs and FNs-Ca(II) 

**Figure 5:**
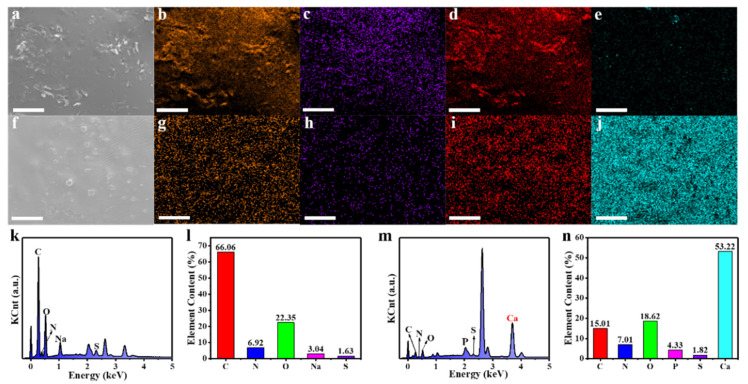
SEM images of FNs (**a**) and FNs-Ca(II) (**f**). Distribution diagram of C (**b**) for FNs and (**g**) for FNs-Ca(II); N (**c**) for FNs and (**h**) for FNs-Ca(II); O (**d**) for FNs and (**i**) for FNs-Ca(II); and Ca (**e**) for FNs and (**j**) for FNs-Ca(II). EDS spectrum of FNs (**k**) and FNs-Ca(II) (**m**), and element statistics of FNs (**l**) and FNs-Ca(II) (**n**). Scale bar is 50 μm.

Energy dispersive X-ray spectroscopy (EDS) has been considered as an effective technique to characterize the elements in a testing sample according to the wavelength of the characteristic X-ray emitted by the sample [32]. In order to confirm the element composition of FNs and FNs-Ca(II), an EDS element analysis was carried out for the FNs and FNs-Ca(II). Figure 5a,f show the scanning electron microscope (SEM) images of the FNs and FNs-Ca(II), respectively. The element surface scan analysis (Figure 5b–d,g–i) showed that there were three main elements (C, N, and O) distributed on the surfaces of the FNs and FNs-Ca(II). Significantly, 53.2% of the Ca element appeared on the surface of FNs-Ca(II) (Figure 5j), while there was less Ca in the FNs (Figure 5e). The EDS spectrum of FNs-Ca(II) showed the specific signal peak of calcium (Ca, 3.7 keV) [33]. Combined with the results of the XPS analysis, these results show that the FNs and FNs-Ca(II) mainly contained three elements (C, N, and O), and the calcium ions were successfully chelated with the FN carriers. 

### 3.6. ^1^H NMR Analysis of FNs and FNs-Ca(II)

**Figure 6:**
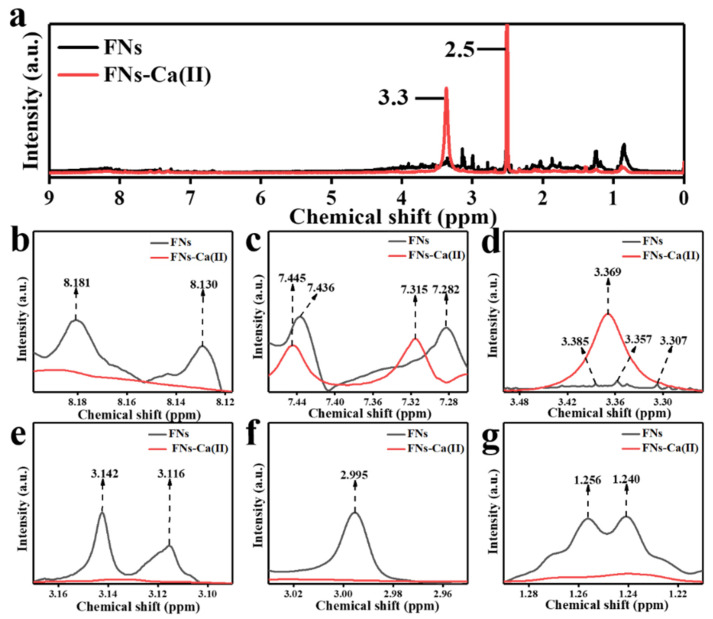
^1^H nuclear magnetic resonance (NMR) spectra of the FNs and FNs-Ca(II) (**a**), and enlarged ^1^H NMR spectra (**b**–**g**) showing the specific NMR peaks of the FNs and FNs-Ca(II).

^1^H NMR is a powerful technique that can be used to analyse the chelation between FNs and calcium ions by measuring chemical shifts [34]. Figure 6a shows a signal at 2.5 ppm that came from the solvent (dimethyl-d_6_ sulfoxide) [35]. Double peaks representing the hydrogen atom of an aromatic ring appeared at 8.181 ppm and 8.130 ppm in the FNs, and disappeared in FNs-Ca(II) (Figure 6b). This may be due to the binding of calcium ions to the aromatic ring of the amino group of FNs [36]. In addition, the signals at 7.282 and 7.436 ppm (Figure 6c) were assigned to the hydrogen signal of -CH in the aromatic compound [37], which shifted to 7.315 and 7.445 ppm after binding with calcium ions. The signals at 3.385, 3.357, and 3.307 ppm were ascribed to methylene in -N-CH_2_ (Figure 6d,e) [38,39,40], which changed from three peaks to one peak after binding with Ca(II). The signals at 3.142 and 3.136 ppm were attributed to the hydrogen atom of -O-C-H [41]. The proton at 2.995 ppm, from a hydroxyl or methylene group adjacent to a nitrogen atom, disappeared in the ^1^H NMR spectrum of FNs-Ca(II) (Figure 6f) [42]. The signals at 1.256 and 1.240 ppm were likely due to a hydrogen in a methyl group. (Figure 6g). The two peaks disappeared after the formation of the FNs-Ca(II) complex [43]. These chemical shifts also showed that Ca(II) was combined with the FNs using the hydroxyl and amino groups for Ca(II) coordination.

### 3.7. Cellular Bio-Distribution and Cytotoxicity of FNs and FNs-Ca(II)

**Figure 7:**
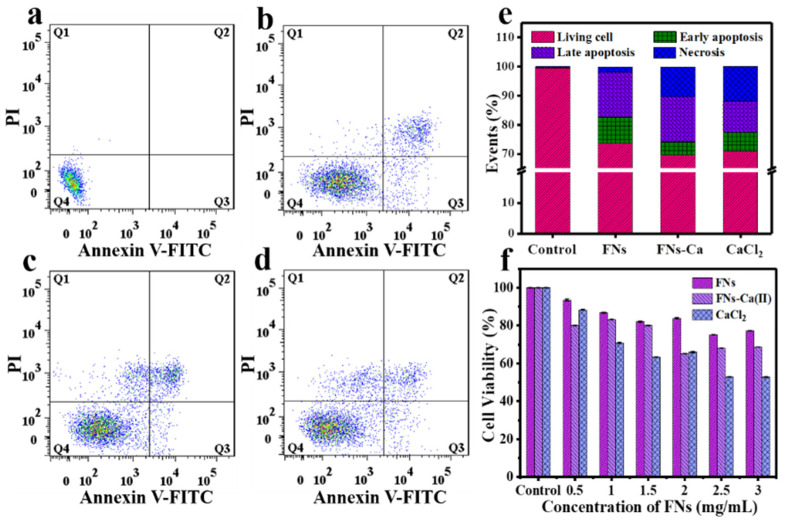
Flow cytometry characterization of Caco-2 cells in control (**a**), 1 mg mL^−1^ of FNs (**b**), 1 mg mL^−1^ of FNs-Ca(II) (**c**), and 1 mg mL^−1^ of CaCl_2_ (**d**). (**e**) Relative apoptosis of cells treated with FNs, FNs-Ca(II), and CaCl_2_. (**f**) Cytotoxicity of Caco-2 cells treated with FNs, FN-Ca(II), and CaCl_2_.

Nanoparticles can cross biological barriers and deliver cargo as carriers to cells [44]. The biocompatibility of FNs-Ca(II) was studied by co-culture with Caco-2 cells. We used annexin V-FITC and PI staining methods to evaluate the cytotoxicity of FNs-Ca(II) (Figure 7). The cell viability was 99.50% in the blank control group, which decreased to 70.7% for the FNs and 69.9% for FNs-Ca(II). The apoptosis rates of FNs-Ca(II) and the FNs were 20.06% and 24.36%, respectively, when they were both at 1 mg mL^−1^. In addition, the necrosis rate of FNs-Ca(II) was 10.30%, compared with a rate of 1.93% for the FNs. It is well all known that almost all cell responses, from contraction and exocytosis to gene expression and cell death, are controlled by changes in Ca(II) concentration in the cytoplasm [45]. After entering the Caco-2 cells, a great deal of calcium ions were released from FNs-Ca(II), which increased the concentration of calcium ions in the cytoplasm and eventually resulted in an increase in cell necrosis rate. In particular, adding free calcium ions resulted in the highest necrosis percentage of 11.9% under the same concentration treatment. Therefore, the FNs can not only be used as carriers for calcium ions, but can also be used to improve the biocompatibility of Ca(II). The influence of FNs-Ca(II) on the percentage of living cells was also evaluated using an MTT assay (Figure 7f). The cytotoxicity assay of the Caco-2 cells showed that the cell viability decreased in all samples when the concentration increased from 0 to 3.0 mg mL^−1^. The viability of the cells treated with FNs was more than 75%, while viability was only 65% after treatment with FNs-Ca(II) and 60% after CaCl_2_ treatment. This biocompatibility of FNs-Ca(II) was improved in contrast to that of CaCl_2_. Compared with the previous nanocarriers that could bind ferrous ions and zinc ions in roast beef patties and beef broth, respectively, we found that the FNs and FNs-Ca(II) had the characteristic of low toxicity [15,26]. These results showed that the FNs-Ca(II) complex has lower cytotoxicity and thus a great potential for application as a carrier for calcium supplements.

**Figure 8:**
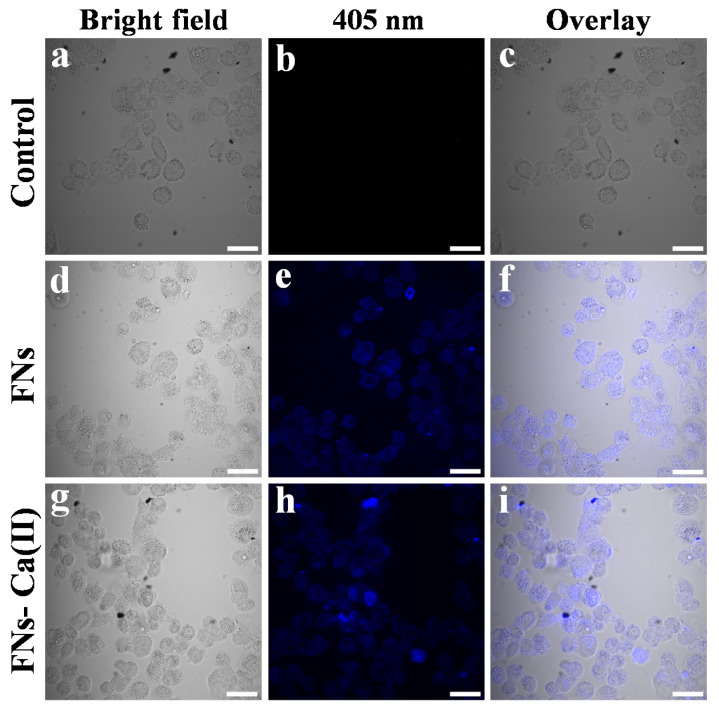
Confocal laser fluorescence images of Caco-2 cells treated with FNs and FNs-Ca(II) with a 408 nm laser. Bright-field images of Caco-2 cells (**a**,**d**,**g**), fluorescence images of Caco-2 cells (**b**,**e**,**h**), and overlay images of Caco-2 cells (**c**,**f**,**i**). Scale bar representing 30 μm.

The unique blue fluorescence properties of the FNs and FNs-Ca(II) were helpful to monitor their distribution in cells. Figure 8 shows the bright-field images and fluorescence images under the irradiation of a 405 nm laser after a 24 h incubation with the FNs and FNs-Ca(II). Blue fluorescence was observed after treatment with FNs and FNs-Ca(II) under an excitation wavelength of 405 nm. It can be seen that most of the FNs and FNs-Ca(II) were distributed in the cytoplasm of the cells instead of in the nucleus (Figure 8e,h). The FNs and FNs-Ca(II) distributed in the Caco-2 cells can be found in the overlay images. The results revealed that the FNs-Ca(II) complex was internalized into the cytoplasm of the Caco-2 cells, which was consistent with the previous observations of nanocarriers in roast beef patties and beef broth [15,26].

## 4. Conclusions

In conclusion, FNs from beef were prepared by green synthesis using the hydrothermal method. The FNs had a small size, blue fluorescence, plenty of functional groups, and good biocompatibility. The functional groups on the surface of the FNs made significant contributions to the chelation of Ca(II), and the amino nitrogen atom and carboxyl oxygen atom were the main binding sites. Our data suggest that the FNs produced from beef might be used for loading calcium in the development of a calcium supplement. The FNs are derived from the nutritional ingredients of beef, which may be desirable for a health-promoting calcium supplement. Meanwhile, FNs-Ca(II) has the potential to be developed into a calcium nanocarrier, and is expected to become a new calcium supplement. The results of this study also provided a new strategy for developing food-borne nanocarriers for other microelement supplements.

## Data Availability

The datasets generated for this study are available on request to the corresponding author.

## Supplementary Materials

The following are available online at https://www.mdpi.com/article/10.3390/foods11030308/s1, Figure S1: (**a**) Determination of calcium binding capacity and (**b**) calcium standard curve.
