# Supplementary figures and images for: Food-Borne Nanocarriers for Calcium Delivery: A New Choice for Nutrient Supplements
Source: Foods. 2022 Jan 24;11(3):308. doi: 10.3390/foods11030308 (PMC8834597; doi:10.3390/foods11030308)

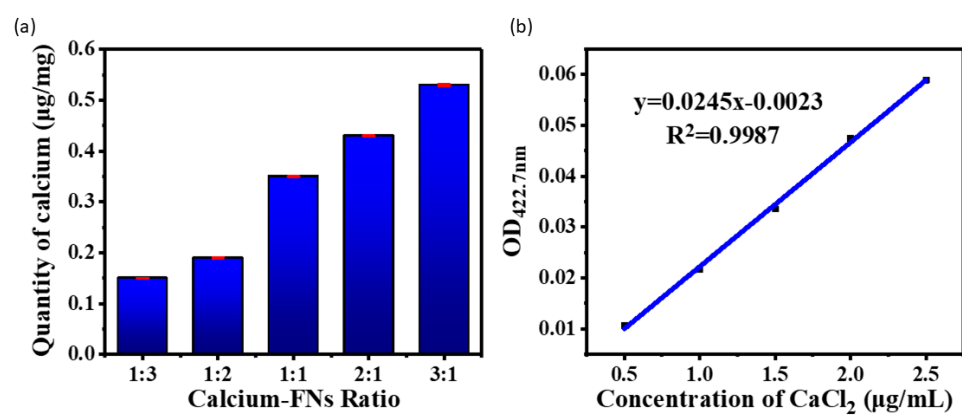

**Figure S1.** (a) Determination of calcium binding capacity and (b) calcium standard curve.

Supplement: Supplementary file 1 [file foods-11-00308-s001.zip › foods-1538464-supplementary.pdf]
